# Role of electrostatic interactions for ligand recognition and specificity of peptide transporters

**DOI:** 10.1186/s12915-015-0167-8

**Published:** 2015-08-06

**Authors:** Rajendra Boggavarapu, Jean-Marc Jeckelmann, Daniel Harder, Zöhre Ucurum, Dimitrios Fotiadis

**Affiliations:** Institute of Biochemistry and Molecular Medicine, and Swiss National Centre of Competence in Research (NCCR) TransCure, University of Bern, Bühlstrasse 28, CH-3012 Bern, Switzerland

**Keywords:** Membrane protein, Peptide transporter, Structure, Three-dimensional crystal, X-ray crystallography, *Yersinia*

## Abstract

**Background:**

Peptide transporters are membrane proteins that mediate the cellular uptake of di- and tripeptides, and of peptidomimetic drugs such as β-lactam antibiotics, antiviral drugs and antineoplastic agents. In spite of their high physiological and pharmaceutical importance, the molecular recognition by these transporters of the amino acid side chains of short peptides and thus the mechanisms for substrate binding and specificity are far from being understood.

**Results:**

The X-ray crystal structure of the peptide transporter YePEPT from the bacterium *Yersinia enterocolitica* together with functional studies have unveiled the molecular bases for recognition, binding and specificity of dipeptides with a charged amino acid residue at the N-terminal position. In wild-type YePEPT, the significant specificity for the dipeptides Asp-Ala and Glu-Ala is defined by electrostatic interaction between the in the structure identified positively charged Lys314 and the negatively charged amino acid side chain of these dipeptides. Mutagenesis of Lys314 into the negatively charged residue Glu allowed tuning of the substrate specificity of YePEPT for the positively charged dipeptide Lys-Ala. Importantly, molecular insights acquired from the prokaryotic peptide transporter YePEPT combined with mutagenesis and functional uptake studies with human PEPT1 expressed in *Xenopus* oocytes also allowed tuning of human PEPT1’s substrate specificity, thus improving our understanding of substrate recognition and specificity of this physiologically and pharmaceutically important peptide transporter.

**Conclusion:**

This study provides the molecular bases for recognition, binding and specificity of peptide transporters for dipeptides with a charged amino acid residue at the N-terminal position.

**Electronic supplementary material:**

The online version of this article (doi:10.1186/s12915-015-0167-8) contains supplementary material, which is available to authorized users.

## Background

Peptide transporters from the proton-dependent oligopeptide transporter (POT) family [1] are integral membrane proteins that mediate the cellular uptake of di- and tripeptides using energy provided by transmembrane proton gradients. POTs belong to the major facilitator superfamily (MFS) of secondary active transporters and are found in all kingdoms of life where they play major roles in nutrition and signaling [2]. In humans the absorption of dietary peptides and of numerous orally administrated drugs is mediated by the two peptide transporters PEPT1 (SLC15A1) and PEPT2 (SLC15A2). PEPT1 and PEPT2 are predominantly expressed in epithelial cells of the small intestine and kidney, respectively [3]. PEPT1 operates as a high-capacity, low-affinity transporter with affinities (K_m_) and inhibition constants (K_i_) ranging from 200 μM to 10 mM depending on the substrate [4]. On the other hand, PEPT2 is a low-capacity, high-affinity transporter with K_m_ and K_i_ values ranging from 5 μM to 500 μM depending on the substrate [4]. These kinetic properties make PEPT1 and PEPT2 ideal for the promiscuous and more specific absorption of a broad range of dietary peptides in the small intestine and in the kidney, respectively. The human PEPT1 and PEPT2 transporters have become of major pharmaceutical importance, as they are responsible for the uptake of β-lactam antibiotics and peptide prodrugs [5].

The first crystal structure of a POT family member, PepT_So_ from the bacterium *Shewanella oneidensis*, was published in 2011 and revealed a novel occluded conformation of an MFS transporter [6]. In the following years several additional structures of prokaryotic peptide transporters were reported: PepT_St_ from *Streptococcus thermophilus* [7, 8]; GkPOT from *Geobacillus kaustophilus* [9]; PepT_So2_ from *Shewanella oneidensis* [10, 11]; and YbgH from *Escherichia coli* (*E. coli*) [12]. All these structures revealed a conserved architecture consisting of 14 transmembrane α-helices (H) with N- and C-terminal six-helix bundles (H1–H6 and H7–H12) and two additional transmembrane α-helices (HA and HB) connecting the two bundles. The structures of GkPOT [9] and PepT_So2_ [10] were obtained in complex with alafosfalin, an antibacterial phosphono dipeptide, and the structures of PepT_St_ [8] and PepT_So2_ [11] in complex with di- and tripeptides, giving first structural insights into the promiscuous substrate recognition of peptide transporters. In spite of the available structural information several key questions remain open. These questions include the molecular bases for the specific recognition of substrates, such as charged dipeptides and of the individual side chains in dipeptides, namely N- versus C-terminal amino acid side chain.

We have solved the X-ray crystal structure of the POT family member YePEPT from *Yersinia enterocolitica* at 3 Å resolution. This structure together with structure-function studies unveiled the molecular basis for recognition of dipeptides with a charged amino acid side chain at the N-terminal position by YePEPT. Importantly, structural and functional insights gained from this prokaryotic peptide transporter were used to perform specific uptake experiments with human PEPT1 expressed in *Xenopus* oocytes. This knowledge helped to extend our current understanding of substrate recognition and specificity in human PEPT1.

## Results and discussion

From bacteria to mammals, peptide transporters from the POT family (also referred to as the peptide transporter (PTR) family [13]) strongly differ in amino acid sequence and protein size. However, three small protein stretches are conserved: the EFxERFxYYG; GxxxADxxxGKxxTIxxxSxxYxxG (PTR2_1); and FSxFYxAINxGSL (PTR2_2) motifs [2]. Residues of these three signature motifs, which are highly conserved in human PEPT1 (hPEPT1) and PEPT2 (hPEPT2), and other higher eukaryotes, are mostly conserved in YePEPT (Additional file 1: Table S1, see also legend to this table for a short description of the three conserved motifs). The conserved EFxERFxYYG, PTR2_2 and PTR2_1 motifs are essential for peptide transport function [2, 14].

For functional and structural studies, the gene of YePEPT was cloned and the protein overexpressed in *E. coli* (Methods).

### Functional characterization of YePEPT

**Fig. 1:**
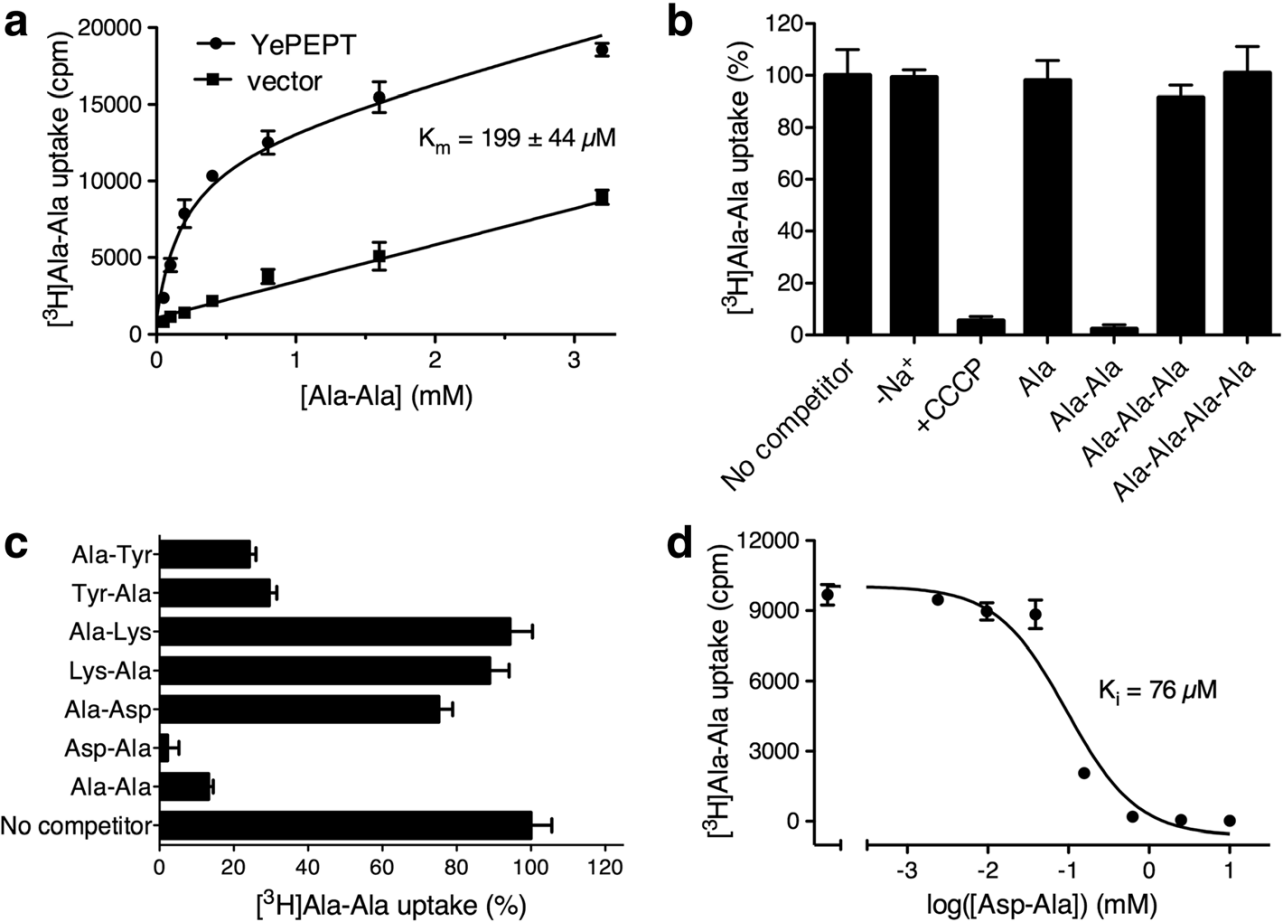
Functional characterization of YePEPT. **a** Kinetics of YePEPT-mediated [^3^H]Ala-Ala uptake in *E. coli* cells. Uptake of the radioligand in *E. coli* cells transformed with the YePEPT construct (YePEPT) and the empty vector (vector; control) is shown. The determined K_m_ is indicated. Error bars represent SEM from triplicates. One representative experiment from three similar independent experiments is shown. **b** Co-transport ion and substrate chain length dependence of uptake: Na^+^ dependence was assessed by replacing Na^+^ with choline (−Na^+^); and H^+^ dependence by addition of the proton-ionophore carbonyl cyanide 3-chlorophenylhydrazone (CCCP). Chain length dependence was assessed with L-Ala and the corresponding di-, tri- and tetrapeptides as competitors (10 mM final concentration). **c** Substrate specificity of YePEPT by competition assay (2.5 mM final concentration). Error bars in (**b**) and (**c**) represent SEM from at least three independent experiments, each in triplicate. **d** K_i_ determination of YePEPT for Asp-Ala. The determined K_i_ is indicated (95 % confidence intervals: 46–126 μM). Error bars represent SEM from triplicates. One representative experiment from three similar independent experiments is shown

Transport function of YePEPT was determined using the reporter radioligand [^3^H]Ala-Ala, which displayed a K_m_ of about 200 μM (Fig. 1a). This K_m_ is comparable to the determined K_i_s of hPEPT1 (about 160 μM) and hPEPT2 (about 100 μM) for Ala-Ala [15]. Competition experiments with [^3^H]Ala-Ala in the presence of the amino acid L-Ala and its di-, tri- and tetrapeptides indicated a clear preference of YePEPT for the dipeptide Ala-Ala only (Fig. 1b). This is in contrast to hPEPT1 and hPEPT2, and numerous other POT family members, which also possess specificity for the tripeptide Ala-Ala-Ala and other tripeptides [2, 15]. For further characterization of transport, we studied the requirements of Na^+^ and H^+^ as co-transport ions. The replacement of Na^+^ by choline had no effect on the uptake of [^3^H]Ala-Ala, but the presence of the proton ionophore carbonyl cyanide 3-chlorophenylhydrazone (CCCP) caused complete abolition of transport (Fig. 1b). Thus, and as expected for POT family members, peptide uptake via YePEPT depends on the electrochemical proton gradient across the membrane.

The dipeptide binding preference of YePEPT (Fig. 1c) was addressed as follows: In a systematic manner, one amino acid in the reporter dipeptide Ala-Ala was replaced at a time at both positions, R1 (N-terminal amino acid) and R2 (C-terminal amino acid), by amino acids of different chemical properties, namely negatively and positively charged, and hydrophobic. YePEPT indicated an important specificity for a dipeptide with a negatively charged amino acid side chain at the R1 position, that is for Asp-Ala (Fig. 1c). This was further confirmed by the determination of the K_i_ of YePEPT for Asp-Ala (Fig. 1d). With a K_i_ of about 80 μM (Asp-Ala), YePEPT had a clearly higher affinity for a dipeptide with a negatively charged amino acid side chain at R1 position compared to Ala-Ala (K_i_ of about 200 μM; Fig. 1a). To support this finding and for comparison with Asp-Ala, the affinity of YePEPT for Glu-Ala was determined, which was about 50 μM (Additional file 2: Figure S1). Considering these K_i_ values, YePEPT had a slightly higher affinity for Glu-Ala compared to Asp-Ala. However, the affinities for these dipeptides harboring negatively charged residues at the R1 position and differing by one methylene group in chain length were not substantially different. Although not so pronounced as for Asp-Ala and Ala-Ala, YePEPT also displayed specificity for Ala-Tyr and Tyr-Ala at comparable levels (Fig. 1c). Such a behavior, that is the indifference for the position of the large, hydrophobic side chain of the amino acid residue Tyr in dipeptides, was also observed for hPEPT1 [16].

### Overall structure of YePEPT

**Fig. 2:**
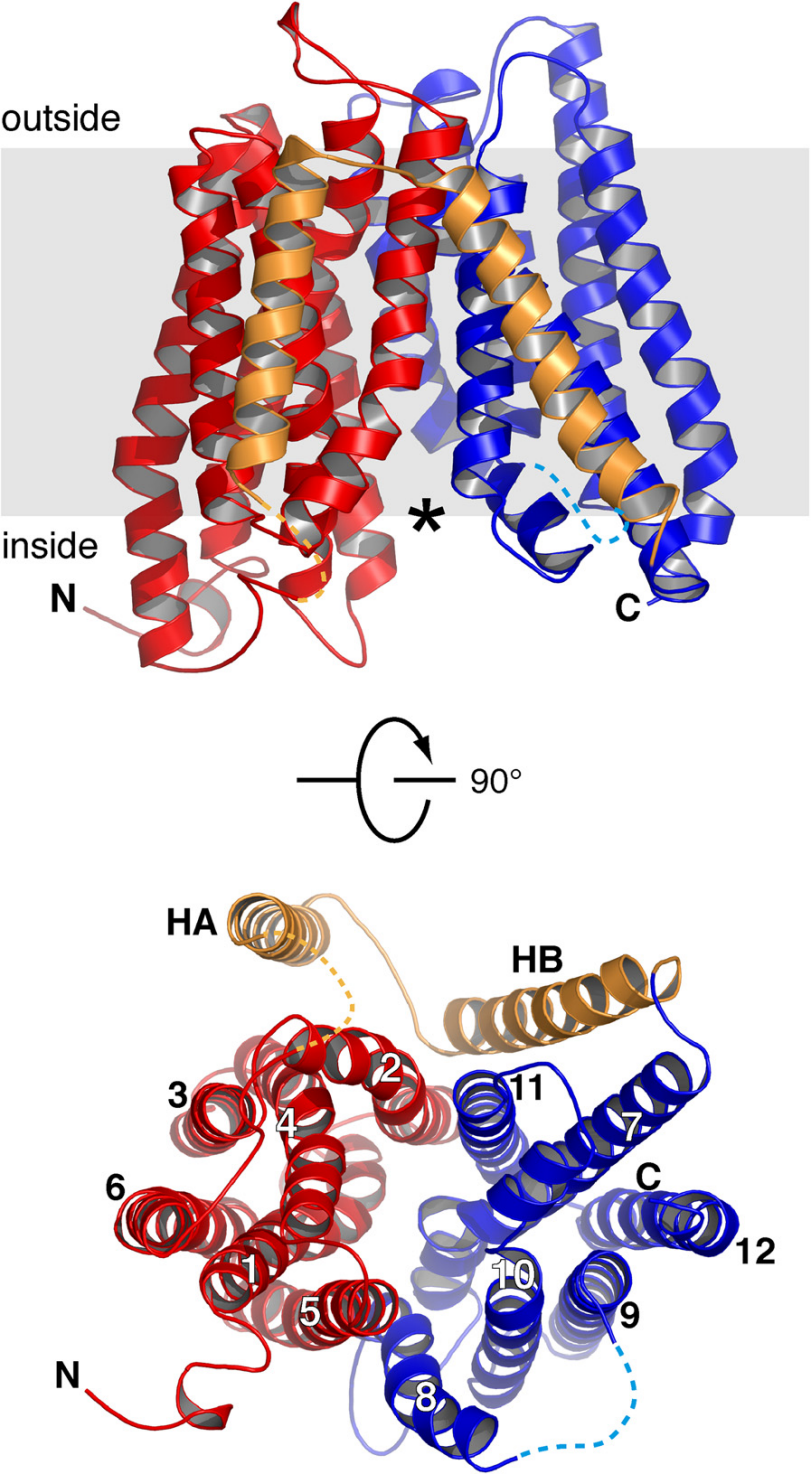
Overall structure of YePEPT. Structure of YePEPT viewed in the plane of the membrane (top) and from the cytosol (bottom). The N- and C-terminal six-helix bundles are colored in red and blue, respectively. The two helices HA and HB connecting the two bundles are colored in gold. The N- and C-termini are labeled. In the top view (bottom) the transmembrane helices H1–H12, and HA and HB are labeled. Parts of the loops connecting H6 and HA, and H8 and H9 that could not be traced are indicated by broken lines

The crystal structure of YePEPT was solved at 3 Å resolution by X-ray crystallography (Methods and Table 1). The quality of the obtained density map can be assessed in Additional file 3: Figure S2. The structure displays 14 transmembrane helices with H1–H6 and H7–H12 forming the N- and C-terminal six-helix bundles (Fig. 2, bundles in red and blue). These two terminal domains are related by a pseudo-twofold symmetry (particularly distinct in Fig. 2, bottom) and are representative of the canonical MFS fold [17]. The two peripheral transmembrane α-helices HA and HB (Fig. 2, in gold) form a hairpin-like structure connecting N- and C-terminal bundles, and are generally found in prokaryotic peptide transporters. The function of these two linker transmembrane α-helices is currently unclear. The solved YePEPT crystal structure is in the inward-open conformation, similar to most prokaryotic peptide transporter structures [7–12] (see also Additional file 4: Figure S3 and its legend for a structural alignment and RMSD values of YePEPT with other peptide transporter structures in the inward-open conformation). This conformational state displays a large central, conical cavity facing the cytosol (Fig. 2, top; asterisk).

**Table 1:** Data collection and refinement statistics for YePEPT

Data collection	
Beamline	X06SA, SLS
Wavelength (Å)	1.00
Space group	P2_1_2_1_2_1_
Unit cell parameters (Å, °)	*a* = 90.7, *b* = 101.0, *c* = 104.2, *α = β = γ =* 90
Resolution (Å)	46.30–3.02 (3.18–3.02)
Number of observed reflections	126,940 (18,201)
Number of unique reflections	19,398 (2,737)
*R*_merge_ (%)	5.6 (99.7)
Completeness (%)	99.8 (98.8)
Multiplicity	6.5 (6.6)
*I*/σ*I*	20.0 (2.0)
CC_1/2_ (%)^a^	99.9 (76.1)
Refinement	
Resolution (Å)	46.30–3.02 (3.10–3.02)
R_work_/R_free_ (%)	25.71 (36.07)/29.40 (39.11)
*B* factor (Å^2^)	105.0
Number of atoms	3,686
RMSD	
Bond length (Å)	0.005
Bond angle (°)	0.889
Ramachandran plot (%)	
Favored region	97.5
Allowed region	100
Disallowed region	0

Values in parentheses reflect the highest resolution shell. ^a^Percentage of correlation between intensities from random half data sets. RMSD, root-mean-square deviation; SLS, Swiss Light Source

### Substrate-binding pocket of YePEPT

**Fig. 3:**
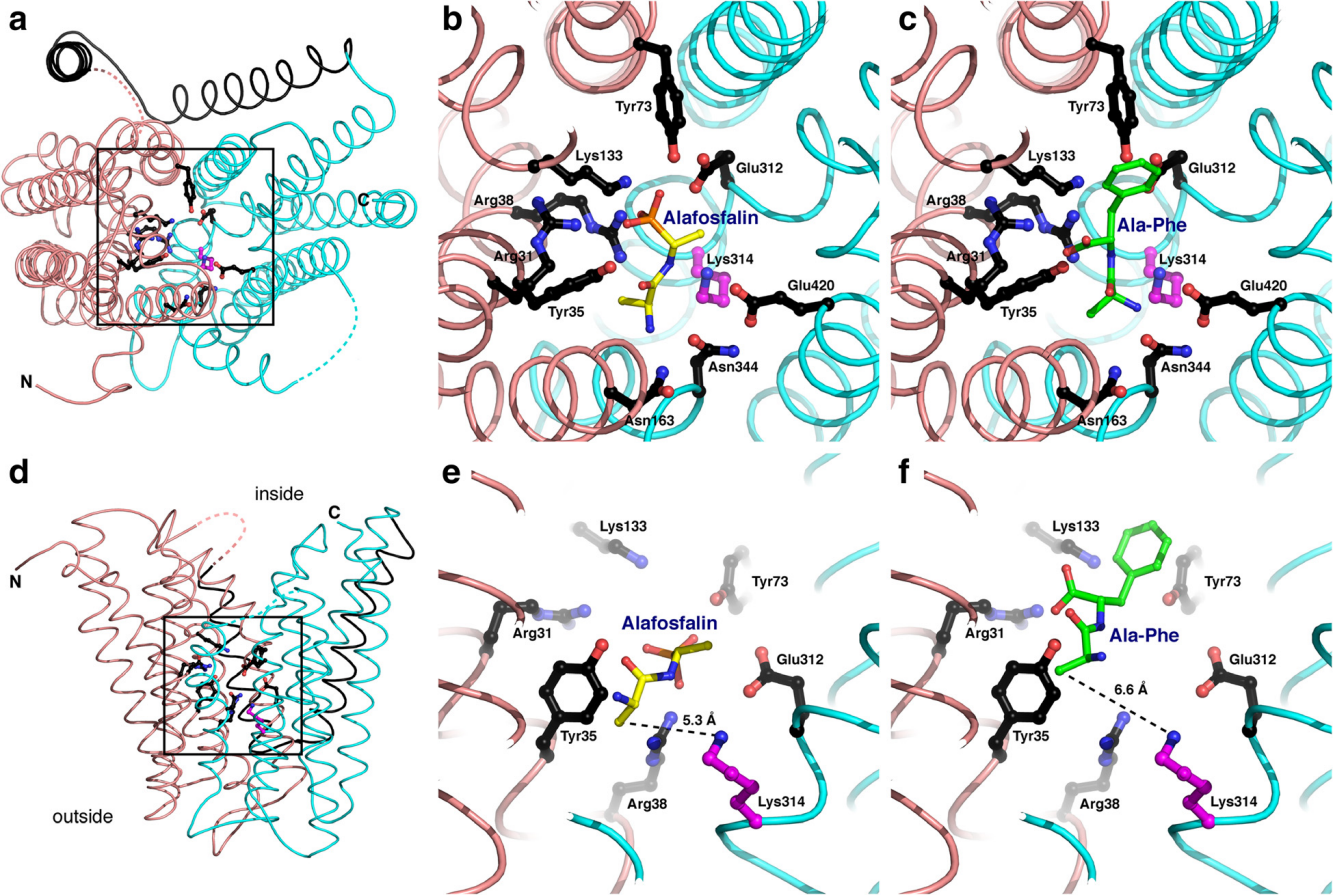
Substrate-binding pocket of YePEPT. Top view from the cytosol (**a**) and view from the membrane plane (**d**) of the substrate-binding pocket of YePEPT. The areas marked by the black boxes in (a) and (d) are displayed at higher magnification in (**b**) and (**c**), and (**e**) and (**f**), respectively. Amino acid residues of YePEPT potentially involved in alafosfalin (in yellow; (b) and (e)) and dipeptide backbone binding (Ala-Phe dipeptide in green; panels (c) and (f)) are labeled, colored in black and conserved in YePEPT, GkPOT^E310Q^ and PepT_St_ (see Additional file 6: Table S2 for more details and groups of conserved amino acid residues involved in alafosfalin and Ala-Phe dipeptide backbone binding). The indicated amino acid residues of YePEPT were identified by superposition of the YePEPT structure with the ligand-bound peptide transporter structures 4D2C [8] and 4IKZ [9] (PDB ID codes). The distances between the nitrogen atom of Lys314 and the carbon atoms of the N-terminally located methyl groups of alafosfalin (e) and Ala-Phe (f) are marked, and indicate available space between ligands and Lys314; for example, for accommodation of longer side chains. The N- and C-terminal six-helix bundles are colored in salmon and cyan, respectively, and helices HA and HB in black. YePEPT polypeptide chains in (a) and (d) that could not be traced are indicated by broken lines

At present the structures of the peptide transporters GkPOT mutant E310Q (GkPOT^E310Q^) [9] and PepT_St_ [8] represent the crystal structures solved in complex with substrates at the highest resolution, namely GkPOT^E310Q^ with alafosfalin at 2.40 Å (PDB ID code: 4IKZ) and PepT_St_ with the dipeptide Ala-Phe at 2.47 Å (PDB ID code: 4D2C). We made numerous attempts to co-crystallize YePEPT with substrate, but were not successful. Therefore, and to identify the putative substrate-binding pocket of YePEPT, we aligned the crystal structure of YePEPT with the substrate-bound GkPOT^E310Q^ and PepT_St_ structures. This yielded hypothetical models of substrate-bound YePEPT. Comparison of these models identified the YePEPT residues located in proximity of the two ligands (Fig. 3; see also Additional file 5: Figure S4 for a comparison of the binding pockets of YePEPT, GkPOT^E310Q^ and PepT_St_). No noteworthy clashes were observed between the YePEPT structure and the ligands alafosfalin and Ala-Phe, the closest interatomic distances being 1.8 Å and 1.5 Å, respectively. All amino acid residues in GkPOT^E310Q^ and PepT_St_ involved in the binding of the backbone of the dipeptide analogue alafosfalin and the dipeptide Ala-Phe were also conserved in YePEPT (see Fig. 3 and Additional file 5: Figure S4: residues colored in black; and Additional file 6: Table S2). These conserved amino acid residues were classified into four groups according to their interactions with the backbones of alafosfalin and Ala-Phe: group i) Asn344 and Glu420 (N-terminal amino group); group ii) Tyr35 and Asn163 (carbonyl group); group iii) Tyr35, Arg38, Tyr73 and Glu312 (phosphonate group); and group iv) Arg31 and Lys133 (C-terminal carboxyl group) (amino acid residues indicated for YePEPT; Fig. 3 and Additional file 6: Table S2). Besides group iii), which specifically interacts with the phosphonate group of alafosfalin, the other three groups represent the general recognition mode of the backbone of dipeptides. These interactions of the transporter with the dipeptide backbone, which are dipeptide side chains independent interactions, can be considered as responsible for the substrate polyspecificity (promiscuity) observed in most peptide transporters.

**Fig. 4:**
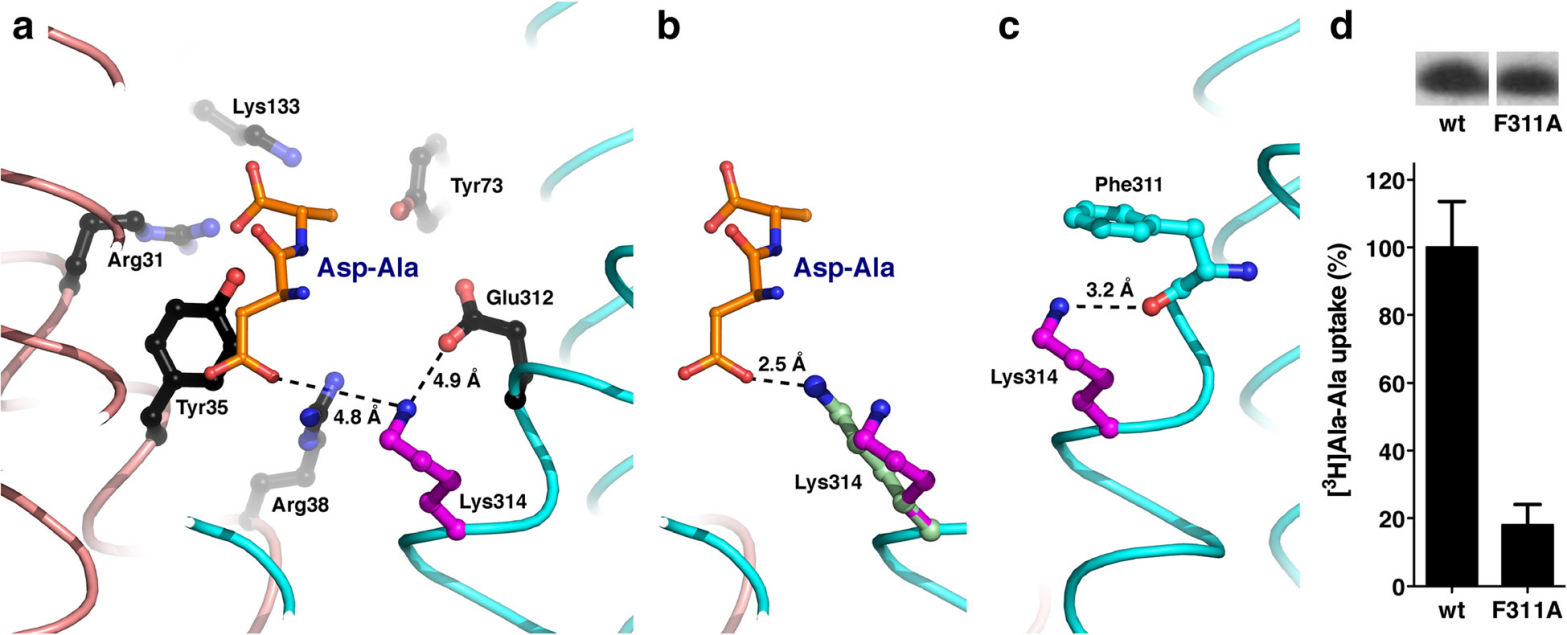
Hypothetical model of Asp-Ala bound YePEPT and stabilizing effect of Phe311. **a** YePEPT structure with a virtually bound Asp-Ala dipeptide (view from the membrane plane): this dipeptide was built by keeping the dipeptide backbone of Ala-Phe (Fig. 3c,f) fixed and mutating the side chains of Ala-Phe into Asp-Ala in Pymol [23]. The rotamer of Asp in Asp-Ala does not introduce any clashes with the YePEPT structure. The distances between the nitrogen atom of Lys314 and the closest oxygen atoms of the carboxyl groups of Asp-Ala (residue at R1 position) and Glu312 are indicated. **b** The rotamer of Lys314 found in the crystal structure (magenta) and of the rotamer with the shortest distance to the closest oxygen atom of the carboxyl group of Asp-Ala (residue at R1 position) is shown (pale green). **c** In the YePEPT crystal structure, the interactions between Lys314 and the stabilizing residue Phe311 consist of a hydrogen bond and a pi-cation interaction between the ε-amino group of Lys314, and the carbonyl and phenyl groups of Phe311, respectively. Amino acid residues of YePEPT potentially involved in dipeptide backbone binding are labeled and colored in black (similar to Fig. 3). The N- and C-terminal six-helix bundles are colored in salmon and cyan, respectively. **d** Mutation of the Lys314 stabilizing Phe311 residue into Ala dramatically reduces the transport function of YePEPT. Expression levels in *E. coli* of wild-type (wt) and YePEPT^F311A^ (F311A) used for the uptake experiments were comparable. Error bars in (d) represent SEM from two independent experiments, each in triplicate

A prominent feature of YePEPT is the presence of a lysine residue (Lys314) close to the above described binding pocket (Fig. 3). The presence of this positively charged residue was particularly interesting considering the high affinity of YePEPT for dipeptides with a negatively charged amino acid at R1 position, namely Asp-Ala (Fig. 1d) and Glu-Ala (Additional file 2: Figure S1). A plausible molecular explanation for the observed substrate affinity is an electrostatic interaction between the negatively charged carboxyl group at the R1 side chains in Asp-Ala and Glu-Ala, and the positively charged residue Lys314 in YePEPT. Mutagenesis *in silico* of Ala-Phe (Fig. 3c,f) into Asp-Ala, keeping the dipeptide backbone of Ala-Phe fixed and mutating the side chains, yielded a hypothetical model of YePEPT with bound Asp-Ala (Fig. 4a,b). The displayed rotamer of Asp in Asp-Ala fits best into the YePEPT structure without introducing any clashes. Based on this model, the distance between the closest oxygen atom (O) of the β-carboxyl group in the Asp side chain of Asp-Ala and the nitrogen atom (N) of the ε-amino group in the Lys314 side chain was 4.8 Å (Fig. 4a). This relatively large distance is not compatible with a salt bridge. However, adoption of a different rotamer of Lys314 upon binding of Asp-Ala would decrease this distance significantly from 4.8 Å to 2.5 Å, thus allowing the formation of a salt bridge (Fig. 4b). Such a salt bridge would neutralize the positive charge on the ε-amino group of Lys314. But how is this charge stabilized in the absence of a negatively charged ligand such as Asp-Ala? To answer this pertinent question, neighboring residues of Lys314 within a distance of ≤4 Å in the crystal structure were considered and Phe311 was identified as an interacting partner. The O of the Phe311 carbonyl group and the N of the ε-amino group of Lys314 are within hydrogen bonding distance (Fig. 4c). Furthermore, this N is 3.5-4.3 Å away from the carbon atoms of the phenyl group of Phe311 and thus compatible with a pi-cation interaction. Therefore, the positive charge of the ε-amino group of Lys314 is potentially stabilized by two molecular interactions with Phe311 in the apo structure of YePEPT. To test this hypothesis, we introduced the mutation F311A (YePEPT^F311A^) and performed [^3^H]Ala-Ala uptake experiments. Clearly, YePEPT^F311A^ was almost not functional compared with wild-type YePEPT in spite of comparable expression levels (Fig. 4d). This result supports the notion that the positively charged ε-amino group of Lys314 has to be stabilized to preserve peptide transport function. Furthermore, it indicates that the hydrogen bond between the ε-amino group of Lys314 and the backbone carbonyl group from the amino acid residue at position 311, for example Ala in YePEPT^F311A^, is not sufficient for stabilization. Therefore, the above mentioned pi-cation interaction seems to be indispensable for charge stabilization. However, we cannot exclude other effects on the transport function of YePEPT upon removal of such a bulky amino acid residue, such as F311. An interaction of Lys314 with the closely localized Glu312 in YePEPT is improbable or weak when considering the distance of 4.9 Å between the corresponding N and O atoms of these two residues (Fig. 4a).

The existence in other bacterial peptide transporters of a Lys residue at the equivalent position of K314 (YePEPT) in their substrate binding pockets was evaluated. The Phe-Lys region (Additional file 1: Table S1), which contains this critical Lys residue, is identical in several members from the large Enterobacteriaceae family; for example, in members from the genus *Yersinia*, *Enterobacter* and *Klebsiella*. In addition to Enterobacteriaceae, the critical Lys residue is also present in less conserved Phe-Lys regions found in other families of the Gammaproteobacteria; for example, in members from the genus *Arenimonas*, *Shewanella* and *Rheinheimera* from the families Xanthomonadaceae, Shewanellaceae and Chromatiaceae.

### Tuning the substrate specificity of YePEPT

**Fig. 5:**
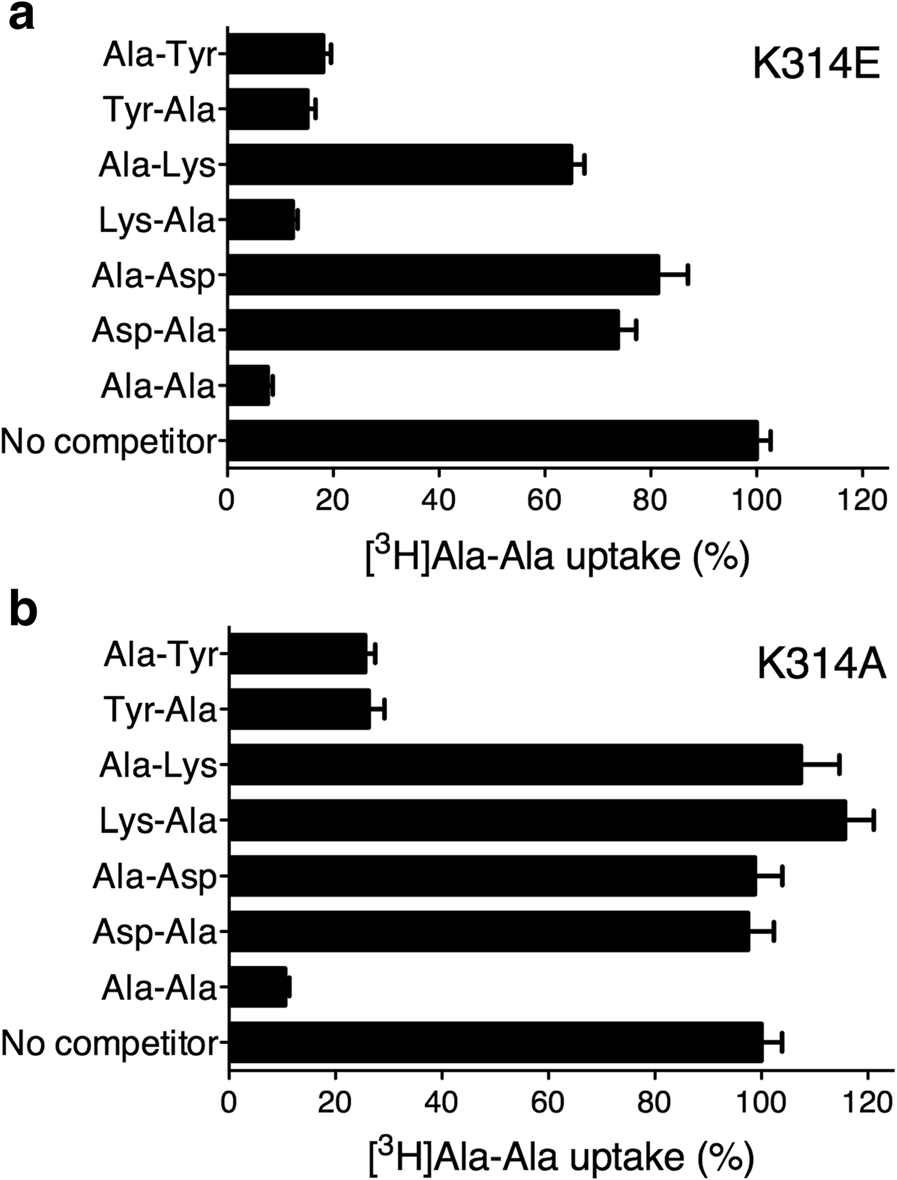
Substrate specificity of K314E and K314A YePEPT mutants. [^3^H]Ala-Ala uptake competition experiments performed with *E. coli* cells expressing (**a**) K314E and (**b**) K314A mutants of YePEPT. Error bars represent SEM from four independent experiments, each in triplicate

From the hypothetical model of Asp-Ala bound YePEPT (Fig. 4), it appears likely that Lys314 is responsible for its specificity for dipeptides with negatively charged amino acids at the R1 position. To test this hypothesis, we introduced the mutation K314E (YePEPT^K314E^) and K314A (YePEPT^K314A^) in YePEPT, and repeated the previous competition experiments. The results clearly demonstrated that the specificity of YePEPT for charged residues could be tuned (YePEPT^K314E^) and abolished (YePEPT^K314A^) in the corresponding mutants (Fig. 5). In more detail, YePEPT^K314E^ acquired specificity for dipeptides with a positively charged amino acid side chain in the R1 position (Lys-Ala; Fig. 5a) and lost the specificity for negatively charged amino acids in that position (Asp-Ala; Fig. 5a). In contrast, YePEPT^K314A^ had no affinity for the charged dipeptides Lys-Ala and Asp-Ala (Fig. 5b). This functional data further supports the notion of the involvement of electrostatic interactions between a charged residue at position 314 of YePEPT and a dipeptide with a charged amino acid side chain at the N-terminal position. An involvement of Lys314 in proton translocation can be excluded because the mutants YePEPT^K314E^ and YePEPT^K314A^ were fully functional (Fig. 5). In contrast to YePEPT, which has a Lys at position 314, GkPOT and PepT_St_ have both a Gly residue at the equivalent position. Based on the results presented above with YePEPT^K314A^, comparable substrate specificities would be expected for GkPOT and PepT_St_. Indeed, GkPOT and PepT_St_ do not show significant specificity for charged dipeptides, but rather specificity for hydrophobic dipeptides [7, 9].

### Influence of the Q300K mutation in human PEPT1 on substrate specificity

**Fig. 6:**
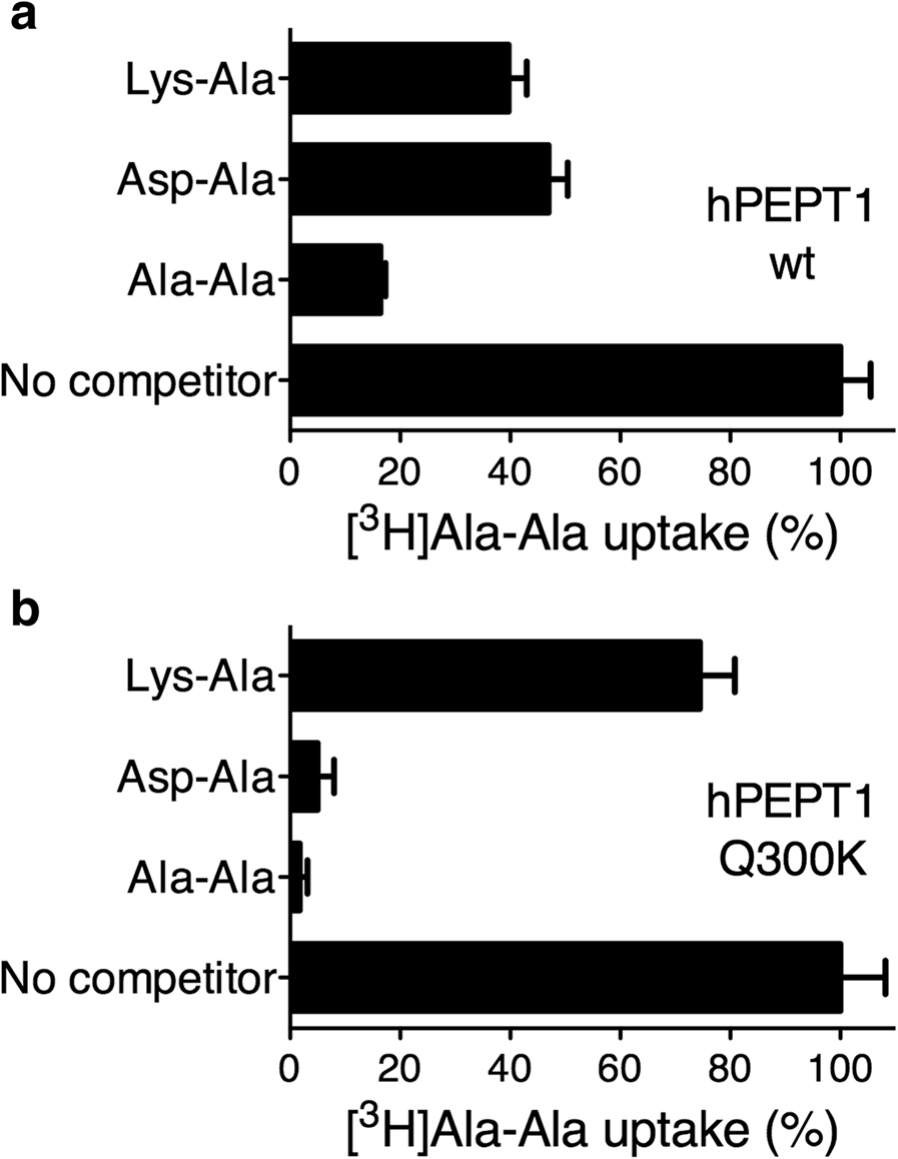
Comparison of substrate specificities of human PEPT1 and Q300K mutant. [^3^H]Ala-Ala uptake competition experiments were performed with *Xenopus laevis* oocytes expressing (**a**) wild-type human PEPT1 (hPEPT1-wt) and (**b**) the Q300K mutant (hPEPT1-Q300K). Error bars represent SEM from two independent experiments, each containing 12 oocytes

In contrast to YePEPT, human PEPT1 does not have a pronounced affinity for dipeptides with a negatively charged amino acid residue at R1 position; for example, Asp-Ala (K_i_ of 320 μM) [18]. Amino acid sequence alignment of YePEPT, hPEPT1 and hPEPT2 identified a Gln residue at the position corresponding to Lys314 in YePEPT (Additional file 1: Table S1, Phe-Lys region). From this alignment it also appears that hPEPT1 has a Phe residue at the position corresponding to Phe311 in YePEPT. The availability of a Phe residue at this critical position in hPEPT1, which we showed to be essential in conjunction with Lys314 in YePEPT for transport function, stimulated us to introduce the mutation Q300K (hPEPT1^Q300K^). Comparison of the specificities for selected substrates in wild-type hPEPT1 and hPEPT1^Q300K^ (Fig. 6) clearly showed that the Q300K mutation induced affinity for Asp-Ala in hPEPT1^Q300K^. Furthermore, the introduction of a positively charged residue in the binding pocket significantly reduced the specificity of hPEPT1^Q300K^ for the positively charged dipeptide Lys-Ala (Fig. 6). In summary, residue 314 in YePEPT corresponds to residue 300 in the binding pocket of hPEPT1. The residue at this critical position determines substrate recognition and specificity for dipeptides charged at R1 position via electrostatic interactions.

## Conclusions

Negatively charged amino acid side chains at the N-terminal position of dipeptides are recognized by Lys314 in YePEPT and interact with each other via electrostatic interactions. Site-directed mutagenesis of Lys314 in YePEPT and the corresponding residue in human PEPT1 (Gln300) can tune the substrate specificity of these peptide transporters. In summary, the crystal structure of YePEPT together with structure-function studies have provided the molecular bases for the recognition, binding and specificity of charged amino acid residues at the N-terminal position of dipeptides by peptide transporters of the POT family.

## Methods

### Cloning of YePEPT

The gene of the peptide transporter YePEPT from *Y. enterocolitica* (UniProt accession number: R9G739) was amplified by PCR from cell lysate (provided by the Vetsuisse Faculty, University of Bern, Bern, Switzerland) using the forward primer 5′-AAAAGCGGCCGCAATGCAAACCTCTACTAAC-3′ and the reverse primer 5′-AAAACTCGAGGGCGGTTTGCTGGCC-3′. The PCR products were digested with the restriction enzymes NotI and XhoI, and ligated into the pZUDF21-rbs-3C-10His expression vector, a pET21 derivative, which produces a target protein with human rhinovirus 3C (HRV3C) protease cleavage site and deca-His tag at the C-terminus [19].

### Protein production and purification

For overexpression, *E. coli* BL21(DE3) pLysS cells were transformed with the pZUDF21-rbs-YePEPT-3C-10His construct. A starter culture was prepared by flushing colonies from the transformation plate into 500 mL of LB medium supplemented with 100 μg/ml ampicillin and then incubated at 37 °C overnight at 180 rpm in an incubation shaker. Next, 20 l LB medium was inoculated 1:100 with the overnight culture. Overexpression was initiated at OD_600_ of 0.8–1.0 by addition of 0.3 mM isopropyl-β-D-1-thiogalactopyranoside and incubated at 20 °C for 16–20 h at 180 rpm in an incubation shaker. Cells were harvested by centrifugation and cell pellets were resuspended in 5 ml lysis buffer (20 mM Tris–HCl, pH 8.0, 150 mM NaCl) per gram of cells. Cells were then lysed using a M-110P Microfluidizer (Microfluidics, Newton, MA, USA) at 15,000 psi pressure during five passages. Cell debris were removed by centrifugation (10,000 × *g*, 20 min, 4 °C). The supernatant was further subjected to ultracentrifugation to collect the membranes (100,000 × *g*, 1 h, 4 °C). Membrane pellets were resuspended in lysis buffer and the previous ultracentrifugation step was repeated. Membranes from 20 l were finally resuspended in 20 ml of resuspension buffer (20 mM Tris–HCl, pH 8.0, 300 mM NaCl) and stored as 1 ml aliquots at −80 °C.

For purification, membranes were solubilized with 2 % (w/v) n-dodecyl-β-D-maltopyranoside (DDM) for 1 h at 4 °C under gentle agitation. Solubilized membranes were separated from unsolubilized fractions by ultracentrifugation (100,000 × *g*, 1 h, 4 °C). The supernatant was diluted 1:1 with binding buffer (20 mM Tris–HCl, pH 8.0, 300 mM NaCl, 40 mM imidazole) and mixed with Ni-NTA Superflow Beads (Qiagen, Limburg, The Netherlands) (0.5 ml bed volume for solubilized membranes from 1 l cell culture), which had been previously equilibrated with equilibration buffer (20 mM Tris–HCl, pH 8.0, 300 mM NaCl, 20 mM imidazole, 0.03 % (w/v) DDM). After 2 h of gentle rotation at 4 °C, the beads were transferred into a column and washed with ten column volumes of washing buffer (20 mM Tris–HCl, pH 8.0, 300 mM NaCl, 5 mM histidine, 0.03 % (w/v) DDM). The column was then equilibrated with five column volumes of cleavage buffer (20 mM Tris–HCl, pH 8.0, 150 mM NaCl, 0.03 % (w/v) DDM) and cleaved on-column by the addition of 250 units of HRV3C protease (BioVision, Milpitas, CA, USA). After incubation for 17 h at 4 °C under gentle rotation, YePEPT was eluted by addition of cleavage buffer.

### Crystallization

Crystallization was performed using a Mosquito robot (TTP Labtech, Melbourn, UK). Drops of 200 nl volume were dispensed in 96-well sitting drop plates and incubated at 18 °C. Initial crystallization hits were obtained with the MemGold (Molecular Dimensions, Newmarket, UK) screen. Best diffracting crystals appeared after 4 days in 25 mM sodium acetate, pH 5.0, 50 mM lithium phosphate, 50 mM lithium sulphate, 32 % PEG300 and a protein concentration of 7 mg/ml. Crystals were harvested, flash-frozen in liquid nitrogen and subjected to X-ray analysis.

### Structure determination and refinement

The native data set of YePEPT was collected at the X06SA (PXI) beamline at Swiss Light Source (SLS; Paul Scherrer Institute, Villigen, Switzerland) and processed with XDS [20]. Phasing by molecular replacement using the coordinates of GkPOT [9] and the following refinement steps were performed using the PHENIX package [21]. Model building was performed using Coot [22]. All structure figures were generated with PyMOL [23]. The data and refinement statistics are listed in Table 1.

### Uptake assay with *E. coli* cells

Precultures of *E. coli* BL21(DE3) pLysS cells containing constructs of wild-type, K314A and K314E of YePEPT, and empty vector (control) were inoculated 1:100 into 80 ml of LB medium with 100 μg/ml ampicillin. Cells were grown in an incubation shaker (37 °C, 180 rpm) and protein expression was induced at an OD_600_ of 0.8–1.0 with 0.3 mM isopropyl-β-D-thiogalactopyranoside. After 3 h of further incubation shaking, cells corresponding to 1 ml at an OD_600_ of 15 were pelleted by centrifugation (5,000 × *g*, 4 °C, 15 min). Cells were then resuspended in 1.5 ml of uptake buffer (50 mM HEPES-NaOH, pH 7.5, 150 mM NaCl, 5 mM glucose). The uptake assay was performed in a final volume of 50 μl per data point, which includes 20 μl of cell suspension, 10 μl of substrate Master mix (yielding a final concentration in the assay of 50 μM Ala-Ala spiked with [^3^H]Ala-Ala (Campro Scientific, Veenendaal, The Netherlands) to a specific activity of 0.04 Ci/mmol) and 20 μl of competitor (yielding a final concentration in the assay of 2.5 mM or 10 mM for the substrate chain length experiment). For K_m_ determination (saturation experiment) various concentrations of [^3^H]Ala-Ala were used at a specific activity of 0.0016 Ci/mmol and 50 μl assay volume. In K_i_ determination experiments various concentrations of Asp-Ala were used as competitor. For Na^+^ dependence experiments, the 150 mM NaCl of the buffer was replaced by 150 mM choline chloride and for H^+^ dependence 50 μM carbonyl cyanide 3-chlorophenylhydrazone (CCCP) was included. Uptake experiments were performed at room temperature under gentle shaking and stopped after 100 s by adding 450 μl of ice-cold uptake buffer. Separation of cells was performed by centrifugation (14,000 × *g*, 2 min). Pellets were resuspended in 50 μl of 5 % (w/v) SDS and transferred to a white 96-well plate (OptiPlate, PerkinElmer, Waltham, MA, USA). Next, 150 μl scintillation cocktail (MicroScint 40, PerkinElmer) were added before measuring with a Packard TopCount (PerkinElmer) scintillation counter. For K_m_ and K_i_ determinations Prism 5 (GraphPad software) was used.

### Uptake assay with *Xenopus* oocytes

For the uptake assay, 10 ng of cRNA coding for human PEPT1 (wild-type) or PEPT1^Q300K^ were injected into *Xenopus laevis* oocytes. Oocytes were incubated at 18 °C for 3 days in Modified Barth’s Medium (MBM; 10 mM HEPES-NaOH, pH 7.4, 88 mM NaCl, 1 mM KCl, 2.4 mM NaHCO_3_, 0.82 mM MgSO_4_, 0.66 mM NaNO_3_, 0.75 mM CaCl_2_) supplemented with 10 μg/ml of penicillin streptomycin antibiotic mixture (Sigma-Aldrich, St Louis, MO, USA). Water-injected oocytes were used as control. Uptake experiments were performed by using pools of 15 oocytes in 2 ml Eppendorf tubes. MBM was carefully removed with a pipette. To the pools of oocytes, 200 μl of the corresponding uptake solution containing 47.5 μM Ala-Ala spiked with 1 μCi of [^3^H]-Ala-Ala (Campro Scientific; final specific activity 0.1 Ci/mmol) and 2.5 mM of competitor (or no competitor) in MBM was added. Mixtures were incubated at room temperature for 15 min with intermittent gentle shaking. Uptake solution was then removed carefully and oocytes were washed four times with 1 ml of cold MBM. Individual oocytes were transferred into wells of a 96-well plate and 50 μl of 5 % (w/v) SDS were added. Plates were shaken at 900 rpm (Eppendorf Thermomixer) until oocytes were completely lysed and the solution looked homogeneous. To each well 150 μl of MicroScint 40 (PerkinElmer) was added followed by mixing (Eppendorf Thermomixer, 500 rpm, 2 min). Plates were measured with a Packard TopCount (PerkinElmer) scintillation counter. Data was analyzed with Prism 5 (GraphPad software).

### Accession codes

Coordinates and structure factors have been deposited in the Protein Data Bank (PDB) under ID code 4W6V.

## Additional files

Additional file 1: Table S1. Conserved motifs in POT/PTR family members and Phe-Lys region. Amino acid sequence alignment was performed with Clustal Omega [24]. The UniProt ID codes of YePEPT, hPEPT1 and hPEPT2 are R9G739, P46059 and Q16348, respectively. The three characters (*, : and ·) indicate positions that have a single, fully conserved residue (*), and conservation between groups of strongly (:) and weakly similar properties (·). The strong and weak groups are defined as strong score >0.5 and weak score ≤0.5 occurring in the Gonnet PAM 250 matrix. Color coding of amino acid residues is according to their physicochemical properties, namely small and hydrophobic (including aromatic except Tyr) (red), acidic (blue), basic (magenta), and other (green) amino acid residues. Phe311 and Lys314 in YePEPT are highlighted in yellow (Phe-Lys region). Short description of the three conserved motifs in POT/PTR family members: The EFxERFxYYG motif is located on H1 and was previously shown to play a role in proton and substrate binding [7–11]. The PTR2_1 motif spans the first cytoplasmic loop that connects H2 and H3, and its function is currently unclear. The PTR2_2 motif is located on H5 and is part of the intracellular gate, which plays a role in regulating the exit of peptides from the substrate-binding site [6]. (DOC 37 kb) Additional file 2: Figure S1. K_i_ determination of YePEPT for Glu-Ala. The determined K_i_ is indicated (95 % confidence intervals: 28–79 μM). Error bars represent SEM from triplicates. One of two similar independent experiments is shown. (TIFF 64 kb) Additional file 3: Figure S2. Electron density map from the YePEPT crystal structure. Stereo view of the final 2 *m*|F_o_|-*D*|F_c_| electron density map of YePEPT after refinement, contoured at 1.0 σ. Helix 1 is shown. The YePEPT structure is depicted by stick models. (TIFF 4156 kb) Additional file 4: Figure S3. Structural alignment of YePEPT with YbgH, GkPOT^E310Q^, PepT_So2_ and PepT_St_. Core structures are displayed in blue (YePEPT), red (YbgH), green (GkPOT^E310Q^), magenta (PepT_So2_) and yellow (PepT_St_). RMSD values are 1.80 Å (for 342 residues; YbgH), 1.73 Å (for 391 residues; GkPOT^E310Q^), 1.78 Å (for 346 residues; PepT_So2_) and 1.51 Å (for 336 residues; PepT_St_). The HA and HB helices were omitted due to their intrinsic flexibility and are only displayed for YePEPT (light grey). PDB ID codes of used models: 4W6V (YePEPT); 4Q65 (YbgH); 4IKZ (GkPOT^E310Q^); 4LEP (PepT_So2_); and 4APS (PepT_St_). (TIFF 4602 kb) Additional file 5: Figure S4. Views on the substrate binding pockets of YePEPT, GkPOT^E310Q^ and PepT_St._ The models of the substrate-free YePEPT and the substrate-bound GkPOT^E310Q^ and PepT_St_ were aligned. For comparison, the binding pockets are shown: YePEPT (A and D); GkPOT^E310Q^ (B and E); and PepT_St_ (C and F). The conserved residues in the binding pockets (see Additional file 6: Table S2 for a detailed description) are shown as black sticks and the substrates are colored in yellow (alafosfalin; GkPOT^E310Q^; B and E) and green (Ala-Phe; PepT_St_; C and F). In all panels the N- and C-terminal six-helix bundles (Cα- backbones) are displayed in reddish and bluish colors, respectively. Note that the positions of the bundles and, most importantly, of the conserved residues are in good agreement. PDB ID codes of used models: 4W6V (YePEPT); 4IKZ (GkPOT^E310Q^); and 4D2C (PepT_St_). (TIFF 5238 kb) Additional file 6: Table S2. Groups of peptide transporter amino acid residues involved and potentially involved in alafosfalin and Ala-Phe dipeptide backbone binding. (DOC 37 kb)

## Abbreviations

CCCP: Carbonyl cyanide 3-chlorophenylhydrazone
DDM: n-dodecyl-β-D-maltopyranoside
MBM: Modified Barth’s Medium
MFS: Major facilitator superfamily
OD: Optical density
PCR: Polymerase chain reaction
PDB: Protein Data Bank
POT: Proton-dependent oligopeptide transporter
PTR: Peptide transporter
RMSD: Root-mean-square deviation
SEM: Standard error of the mean
wt: Wild-type
