# Supplementary material for: Role of electrostatic interactions for ligand recognition and specificity of peptide transporters
Source: BMC Biol. 2015 Aug 6;13:58. doi: 10.1186/s12915-015-0167-8 (PMC4527243; doi:10.1186/s12915-015-0167-8)
Supplement: Additional file 1: Table S1. — Conserved motifs in POT/PTR family members and Phe-Lys region. Amino acid sequence alignment was performed with Clustal Omega [24]. The UniProt ID codes of YePEPT, hPEPT1 and hPEPT2 are R9G739, P46059 and Q16348, respectively. The three characters (*, : and ·) indicate positions that have a single, fully conserved residue (*), and conservation between groups of strongly (:) and weakly similar properties (·). The strong and weak groups are defined as strong score >0.5 and weak score ≤0.5 occurring in the Gonnet PAM 250 matrix. Color coding of amino acid residues is according to their physicochemical properties, namely small and hydrophobic (including aromatic except Tyr) (red), acidic (blue), basic (magenta), and other (green) amino acid residues. Phe311 and Lys314 in YePEPT are highlighted in yellow (Phe-Lys region). Short description of the three conserved motifs in POT/PTR family members: The EFxERFxYYG motif is located on H1 and was previously shown to play a role in proton and substrate binding [7–11]. The PTR2_1 motif spans the first cytoplasmic loop that connects H2 and H3, and its function is currently unclear. The PTR2_2 motif is located on H5 and is part of the intracellular gate, which plays a role in regulating the exit of peptides from the substrate-binding site [6]. (DOC 37 kb) [file 12915_2015_167_MOESM1_ESM.doc]

**Table S1.**  Conserved motifs in POT/PTR family members and Phe-Lys region

| **EFxERFxYYG motif**  YePEPT 27-EMWERFSFYG-36  hPEPT1 23-EFCERFSYYG-32  hPEPT2 53-EFCERFSYYG-62  *: ****:**  motif EFxERFxYYG |
| --- |
| **PTR2_1 motif**  YePEPT 79-GGLLADNWLGQQRAVWYGSILIALG-103  hPEPT1 70-GALIADSWLGKFKTIVSLSIVYTIG-94  hPEPT2 100-GAAIADSWLGKFKTIIYLSLVYVLG-124  *. :**.***: ::: *:: .:*  motif GxxxADxxxGKxxTIxxxSxxYxxG |
| **PTR2_2 motif**  YePEPT 155-FSLFYMGINMGSF-167  hPEPT1 163-FSIFYLAINAGSL-175  hPEPT2 184-FSVFYLSINAGSL-196  **:**:.** **:  motif FSxFYxAINxGSL  **Phe-Lys region**  YePEPT 310-AFEQKPTSFNLFA-322  hPEPT1 296-LFDQQGSRWTLQA-308  hPEPT2 315-LLDQQGSRWTLQA-327  ::*: : :.* * |

Amino acid sequence alignment was performed with Clustal Omega . The UniProt ID codes of YePEPT, hPEPT1 and hPEPT2 are R9G739, P46059 and Q16348, respectively. The three characters (*, : and ·) indicate positions that have a single, fully conserved residue (*), and conservation between groups of strongly (:) and weakly similar properties (·). The strong and weak groups are defined as strong score >0.5 and weak score ≤0.5 occurring in the Gonnet PAM 250 matrix. Color coding of amino acid residues is according to their physicochemical properties, i.e., small and hydrophobic (including aromatic except Tyr) (red), acidic (blue), basic (magenta) and other (green) amino acid residues. Phe311 and Lys314 in YePEPT are highlighted in yellow (Phe-Lys region). Short description of the three conserved motifs in POT/PTR family members: The EFxERFxYYG motif is located on H1 and was previously shown to play a role in proton and substrate binding [7-11]. The PTR2_1 motif spans the first cytoplasmic loop that connects H2 and H3, and its function is currently unclear. The PTR2_2 motif is located on H5 and is part of the intracellular gate, which plays a role in regulating the exit of peptides from the substrate-binding site [6].
