# Supplementary figures and images for: Role of electrostatic interactions for ligand recognition and specificity of peptide transporters
Source: BMC Biol. 2015 Aug 6;13:58. doi: 10.1186/s12915-015-0167-8 (PMC4527243; doi:10.1186/s12915-015-0167-8)

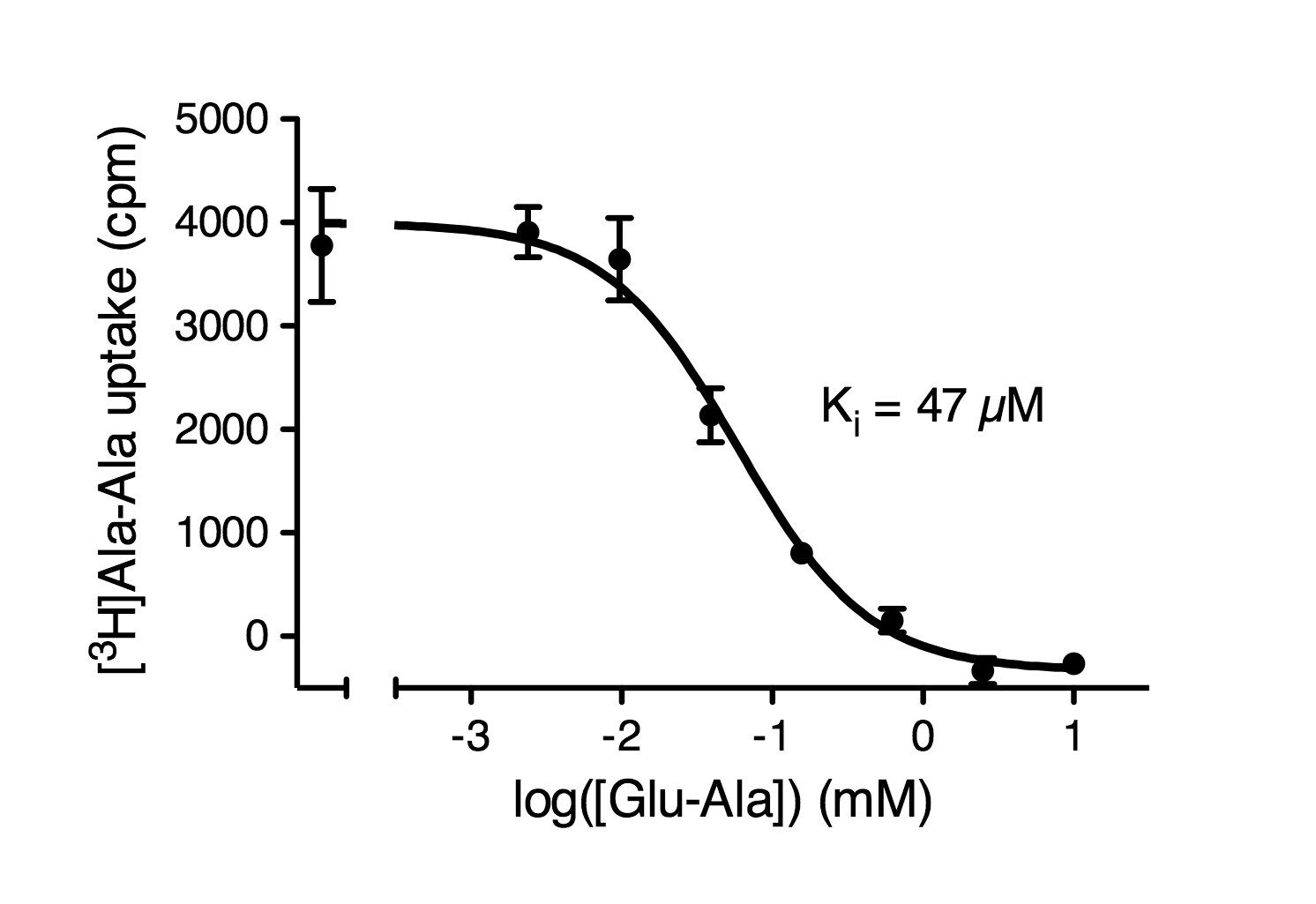

Supplement: Additional file 2: Figure S1. — Ki determination of YePEPT for Glu-Ala. The determined Ki is indicated (95 % confidence intervals: 28–79 μM). Error bars represent SEM from triplicates. One of two similar independent experiments is shown. (TIFF 64 kb) [file 12915_2015_167_MOESM2_ESM.tif]

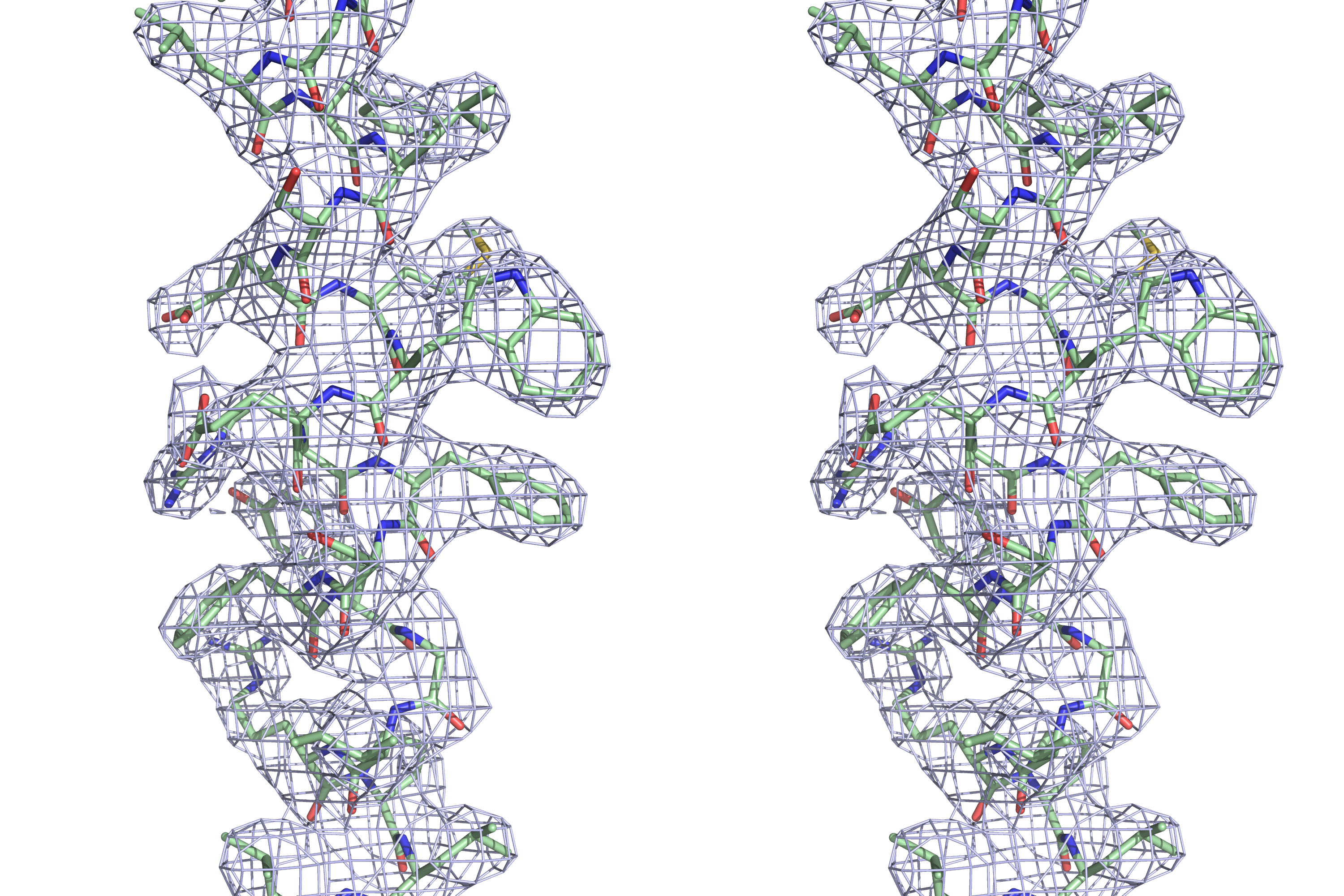

Supplement: Additional file 3: Figure S2. — Electron density map from the YePEPT crystal structure. Stereo view of the final 2 m|Fo|-D|Fc| electron density map of YePEPT after refinement, contoured at 1.0 σ. Helix 1 is shown. The YePEPT structure is depicted by stick models. (TIFF 4156 kb) [file 12915_2015_167_MOESM3_ESM.tif]

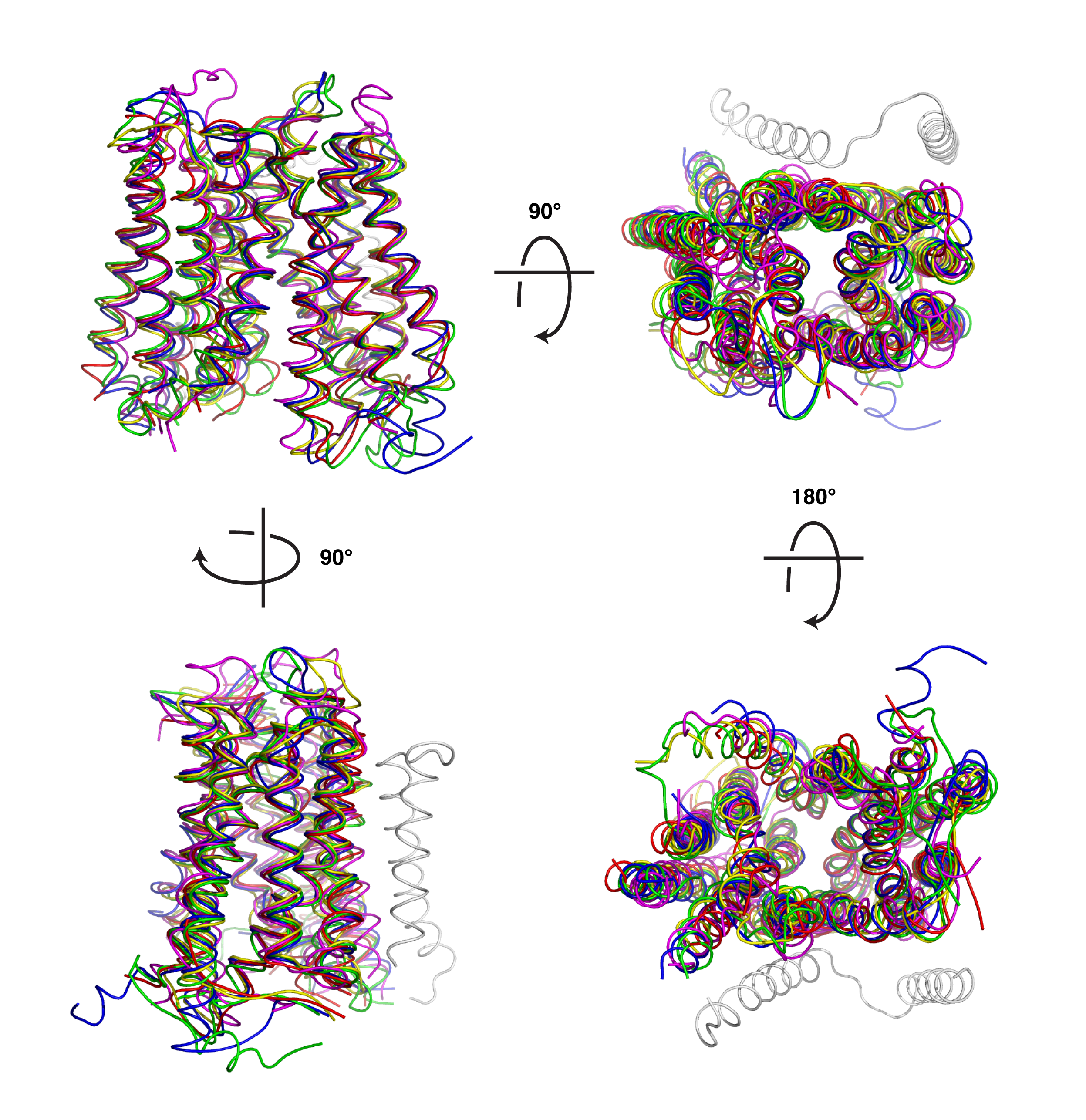

Supplement: Additional file 4: Figure S3. — Structural alignment of YePEPT with YbgH, GkPOTE310Q, PepTSo2 and PepTSt. Core structures are displayed in blue (YePEPT), red (YbgH), green (GkPOTE310Q), magenta (PepTSo2) and yellow (PepTSt). RMSD values are 1.80 Å (for 342 residues; YbgH), 1.73 Å (for 391 residues; GkPOTE310Q), 1.78 Å (for 346 residues; PepTSo2) and 1.51 Å (for 336 residues; PepTSt). The HA and HB helices were omitted due to their intrinsic flexibility and are only displayed for YePEPT (light grey). PDB ID codes of used models: 4W6V (YePEPT); 4Q65 (YbgH); 4IKZ (GkPOTE310Q); 4LEP (PepTSo2); and 4APS (PepTSt). (TIFF 4602 kb) [file 12915_2015_167_MOESM4_ESM.tif]

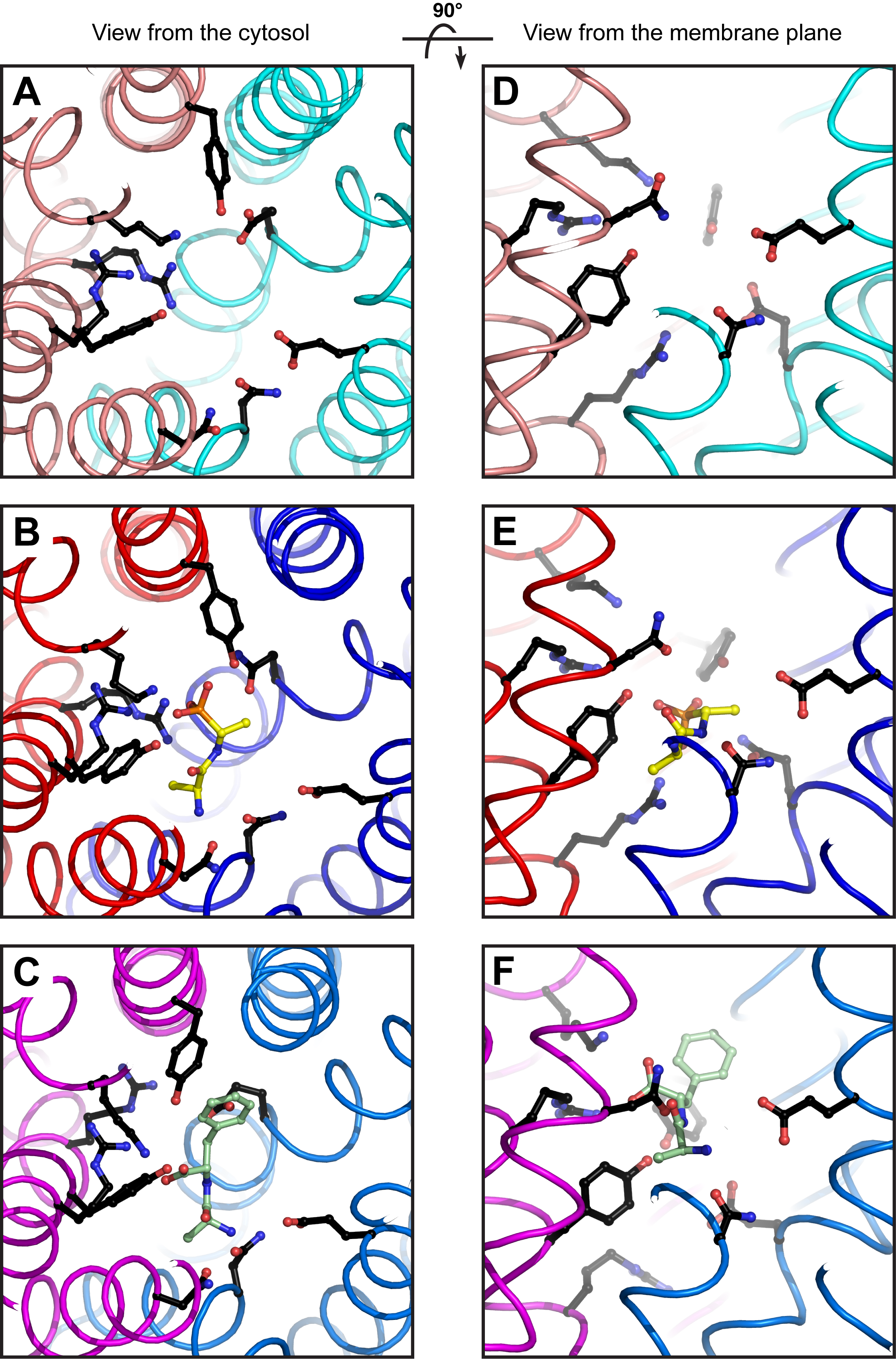

Supplement: Additional file 5: Figure S4. — Views on the substrate binding pockets of YePEPT, GkPOTE310Q and PepTSt. The models of the substrate-free YePEPT and the substrate-bound GkPOTE310Q and PepTSt were aligned. For comparison, the binding pockets are shown: YePEPT (A and D); GkPOTE310Q (B and E); and PepTSt (C and F). The conserved residues in the binding pockets (see Additional file 6: Table S2 for a detailed description) are shown as black sticks and the substrates are colored in yellow (alafosfalin; GkPOTE310Q; B and E) and green (Ala-Phe; PepTSt; C and F). In all panels the N- and C-terminal six-helix bundles (Cα- backbones) are displayed in reddish and bluish colors, respectively. Note that the positions of the bundles and, most importantly, of the conserved residues are in good agreement. PDB ID codes of used models: 4W6V (YePEPT); 4IKZ (GkPOTE310Q); and 4D2C (PepTSt). (TIFF 5238 kb) [file 12915_2015_167_MOESM5_ESM.tif]
