# Supplementary material for: Role of electrostatic interactions for ligand recognition and specificity of peptide transporters
Source: BMC Biol. 2015 Aug 6;13:58. doi: 10.1186/s12915-015-0167-8 (PMC4527243; doi:10.1186/s12915-015-0167-8)
Supplement: Additional file 6: Table S2. — Groups of peptide transporter amino acid residues involved and potentially involved in alafosfalin and Ala-Phe dipeptide backbone binding. (DOC 37 kb) [file 12915_2015_167_MOESM6_ESM.doc]

**Table S2.**  Groups of peptide transporter amino acid residues involved and potentially involved in alafosfalin and Ala-Phe dipeptide backbone binding

| **YePEPT**  4W6V^a^ | **GkPOT^E310Q^**  **alafosfalin**  4IKZ^a^ | **PepT_St_**  **Ala-Phe**  4D2C^a^ | **Helix** |
| --- | --- | --- | --- |
| **Group *i.)***:  Interaction with the N-terminal amino-group of alafosfalin and Ala-Phe dipeptide backbone | | | |
| Asn344 | Asn342 | Asn328 | H8 |
| Glu420 | Glu413 | Glu400 | H10 |
| **Group *ii.)***:  Interaction with carbonyl group of alafosfalin and Ala-Phe dipeptide backbone | | | |
| Tyr35^c^ | Tyr40 | Tyr30 | H1 |
| Asn163 | Asn166 | Asn156 | H5 |
| **Group *iii.)***:  Interaction with the phosphonate group of alafosfalin | | | |
| Tyr35^c^ | Tyr40 | Tyr30 | H1 |
| Arg38 | Arg43 | Arg33 | H1 |
| Tyr73 | Tyr78 | Tyr68 | H2 |
| Glu312 | Gln310 ^b^ | Glu300 | H7 |
| **Group *iv.)***:  Interaction with the C-terminal carboxyl-group of Ala-Phe dipeptide backbone | | | |
| Arg31^c^ | Arg36 | Arg26 | H1 |
| Lys133 | Lys136 | Lys126 | H4 |

^a^ Protein Data bank (PDB) ID code.

^b^ Glu in wild-type (similar to YePEPT and PepT_St_).

^c^ Amino acid residue conserved in EFxERFxYYG motif.
